# Hippocampal and prefrontal GABA and glutamate concentration contribute to component processes of working memory in aging

**DOI:** 10.1093/cercor/bhaf105

**Published:** 2025-05-12

**Authors:** Pernilla Andersson, Xin Li, Jonas Persson

**Affiliations:** Center for Life-span Developmental Research (LEADER), School of Behavioral, Social and Legal Sciences, Örebro University, Fakultetsgatan 1, 702 81, Örebro, Sweden; Aging Research Center (ARC), Karolinska Institute and Stockholm University, Tomtebodavägen 18a, 171 65, Solna, Sweden; Computational Brain Imaging Group, National University of Singapore, 21 Lower Kent Ridge Rd, 119077, Singapore; Center for Life-span Developmental Research (LEADER), School of Behavioral, Social and Legal Sciences, Örebro University, Fakultetsgatan 1, 702 81, Örebro, Sweden; Aging Research Center (ARC), Karolinska Institute and Stockholm University, Tomtebodavägen 18a, 171 65, Solna, Sweden

**Keywords:** aging, GABA, glutamate, spectroscopy, working memory

## Abstract

Both animal and human studies indicate that individual variation in the neurometabolites gamma-aminobutyric acid and glutamate is linked to cognitive function. Age-related differences in these neurometabolites could potentially explain lower cognitive ability in older age. Working memory—the capacity to hold a limited amount of information online for a short period—has a central role in cognition, and this ability is also impaired in older individuals. Here, we investigated the relationship between gamma-aminobutyric acid (GABA^+^) levels and a composite measure of glutamate/glutamine (Glx) in the hippocampus and inferior frontal gyrus (IFG) and how these neurochemical markers relate to working memory in younger and older adults. Across age groups, we found a significant positive association between working memory accuracy and Glx in the IFG, as well as a significant negative association between GABA^+^ in this region and proactive interference. Age-stratified analyses demonstrated significant positive associations between components of working memory and hippocampal/IFG Glx, as well as a significant negative association between IFG GABA^+^ and proactive interference in older adults only. These results provide novel evidence for a specific involvement of excitatory Glx and working memory accuracy as well as inhibitory GABA^+^ for control of proactive interference in working memory, and how these effects are differentially affected by age.

## Introduction

Working memory (WM) capacity—the ability to maintain and manipulate limited information over short timespans—has a central role in human cognition. Individual differences in WM capacity have been proposed to underlie individual differences in general cognitive abilities (Conway et al. 2003; Salthouse and Pink 2008) and have been related to cognitive decline in aging (Naveh-Benjamin and Cowan 2023). Numerous studies have demonstrated age-related impairments in both WM capacity and WM sub-processes, such as interference control (Lustig and Jantz 2014; Ziaei et al. 2017; Samrani and Persson 2021), WM manipulation (Nyberg et al. 2014), and updating (Van der Linden et al. 1994; De Beni and Palladino 2004). Understanding the neural mechanisms of WM would elevate our understanding of WM-dependent cognitive and behavioral capabilities, as well as provide insights into the pathophysiology and remediation of WM deficits in pathological states.

Magnetic resonance spectroscopy (MRS) enables in vivo quantification of metabolites. Of particular interest in previous research has been the measurement of the neurotransmitters glutamate and gamma-aminobutyric acid (GABA). Glutamate is a key molecule in cellular metabolism and the major excitatory neurotransmitter in the central nervous system. Several studies have shown that glutamate is reduced in normal aging (Schubert et al. 2004; Chang et al. 2009; Roalf et al. 2020), although other studies have also failed to find age-related differences in glutamate concentration in some examined regions (Zahr et al. 2008; Nikolova et al. 2017). GABA is the most abundant inhibitory neurotransmitter in the brain and is critical for central nervous system function (Buzsáki et al. 2007). Studies that include samples of middle-aged and older adults typically show an age-related reduction in GABA levels (Gao et al. 2013; Porges et al. 2017a). Recently, it has been demonstrated that the trajectory of GABA across the lifespan may have a nonlinear asymmetric association with age; in childhood and adolescence, the relationship with age is positive, with a gradual decrease starting from middle age into older age (Porges et al. 2021). However, an age-related reduction in GABA with advancing age has not been uniformly demonstrated (Steel et al. 2020).

Both glutamate and GABA are neurochemicals that are critical for cognitive functions, and studies report associations with general cognition in both younger (Yoon et al. 2016; Stanley et al. 2017; Li et al. 2022) and older (Zahr et al. 2008; Nikolova et al. 2017; Porges et al. 2017a) adults, although these relationships are not uniformly demonstrated (Li et al. 2022). Within the memory domain, hippocampal (HC) glutamate has been associated with faster associative learning in younger adults (Stanley et al. 2017) and better word list recall in older, but not younger, adults (Nikolova et al. 2017). Similarly, HC GABA concentration has been linked to better associative learning in healthy younger (Spurny et al. 2020) and older adults (Jiménez-Balado et al. 2021). Moreover, HC GABA concentration has been linked to the ability to suppress no longer relevant memory representations using the think/no-think paradigm (Schmitz et al. 2017).

With regard to WM, animal work has demonstrated that blocking glutamate receptors impairs spatial WM (Morris et al. 1986), and previous human studies have demonstrated a direct link between glutamate concentration and WM using MRS (Rmus et al. 2023), or between WM performance and GABA/glutamate (Takei et al. 2016) and GABA/Glutamate ratio (Marsman et al. 2017). It has also been shown that GABA levels in the dorsolateral prefrontal cortex are linked to WM performance in a sample of younger adults (Yoon et al. 2016). More specifically, individuals with lower GABA levels performed worse on trials with a higher WM load (compared to trials with low load). Additionally, frontal GABA has been associated with better WM in healthy younger adults, while the reverse pattern was found in patients with schizophrenia (Ragland et al. 2020).

A central aspect of WM is updating memory representations into the focus of attention and simultaneously controlling interference from no longer relevant information (Samrani and Persson 2022). Therefore, successful WM performance relies heavily on the ability to resolve competition from different memory representations. Thus, glutamate might be specifically critical for successfully keeping target information in WM, while GABA might be specifically involved in resolving interference from competing and irrelevant information through its involvement in inhibitory functions. Both the maintenance of WM representations and the ability to resolve interference in WM are compromised in older age (Fabiani et al. 2016; Samrani et al. 2017; Samrani and Persson 2021). One contributing factor to this age-related difference could be alterations in neurochemicals such as glutamate and GABA, but these neurobiological underpinnings of age differences in component processes of WM have not yet been investigated.

In the current study, we investigate the contributions of frontal and HC glutamate and GABA levels to different aspects of WM in younger and older adults. We used a 2-back WM task designed to measure the unique contributions of two components: target maintenance and control of proactive interference (PI). These two components are likely supported by distinct neurobiological mechanisms with potentially differential GABA/Glutamate dependencies. Participants were scanned with proton MRS (^1^H MRS) using the MEGA-PRESS sequence to estimate levels of Glx (the combination of glutamate and glutamine) and GABA^+^ (GABA along with potential contributions from co-edited macromolecule signals). Tissue-corrected levels of Glx and GABA^+^ were subsequently investigated in relation to performance on WM component processes across all participants, along with analyses to estimate whether these associations were moderated by age.

## Materials and methods

### Participants

The initial sample consisted of 50 younger and 38 older adults from the local Örebro community in Sweden as part of the Memory, Brain, and Aging project. All participants were native Swedish speakers, right-handed, physically and psychologically healthy, not taking vascular or psychotropic medication, had normal or corrected-to-normal vision, and had no other magnetic resonance imaging (MRI) contraindications. Six younger adults and three older adults did not have complete MRS data, and one older participant scored below 24 on the Montreal Cognitive Assessment (MoCA) and was therefore excluded. An outlier analysis was performed using the interquartile range (IQR; quartile 3—quartile 1) rule of IQR × 3 for detecting the presence of outliers. The outlier analysis was performed for each age group separately. In total, 3 older adults and 2 younger adults were excluded, leaving a final sample of 42 younger adults (mean age = 23.3 years, range = 20—28, female = 23) and 31 older adults (mean age = 73.9 years, range = 65-83, female = 15) for the analyses on relationships between WM performance and GABA (Table 1). All included participants scored 24 or above on the MoCA. All participants received 800 SEK (approximately $75) as compensation for participation. The study was approved by the Swedish Ethical Review Authority (dnr 2020-05299 and dnr 2022-06576-02), and written consent was obtained from all participants.

**Table 1:** Demographic, cognitive, and spectroscopy measurements.

Younger adults		Older adults	Sig. (*p*-values)
N	42	31	
Age (range)	23.3 (20–28)	73.9 (65–83)	**<.001**
Sex (F/M)	23/19	15/16	= 0.79
Education [years]; (SD)	15 (1.9)	14.2 (3.32)	= 0.29
MoCA (std)	28.5 (0.91)	27.3 (1.77)	**=.001**
WM target accuracy (SD)	93.2 (8.41)	82.1 (16.2)	**<.001**
WM target RT (SD)	908 (161)	1154 (227)	**<.001**
PI RT (SD)	236.3 (174.9)	275.9 (264.7)	= 0.88
PI accuracy (SD)	13.8 (11.9)	33.6 (19.3)	**<.001**
PI combined (SD)	−.26 (.54)	0.35 (0.91)	**=.003**
IFG TISSUE – % GM (SD)	57.4 (3.6)	46.7 (4.3)	**<.001**
IFG TISSUE – % WM (SD)	29.6 (4.9)	30.7 (6.5)	=.06
IFG TISSUE – % CSF (SD)	13 (4.4)	21.1 (4.9)	**<.001**
IFG LINEWIDTH SD)	9.24 (1.91)	9.18 (1.2)	= 0.88
IFG SNR (SD)	69.6 (14.6)	64.8 (9.71)	= 0.12
HC TISSUE – % GM (SD)	50.3 (2.7)	45.8 (3.1)	**<.001**
HC TISSUE – % WM (SD)	42.7 (3.1)	39.5 (6.1)	**=.005**
HC TISSUE – % CSF (SD)	6.9 (2.4)	14.6 (6.1)	**<.001**
HC LINEWIDTH (SD)	15.5 (13.6)	14.3 (3.47)	= 0.65
HC SNR (SD)	39.7 (12.6)	33.5 (6.56)	**= 0.016**

SD = standard deviation; MoCA = Montreal Cognitive Assessment; RT = reaction time (in milliseconds); WM = working memory; PI = Proactive interference; IFG = inferior frontal gyrus; HC = hippocampus; GM = gray matter; WM = white matter; CSF = cerebrospinal fluid; SNR = signal-to-noise ratio. Significant *p*-values (*p* < .05) are indicated in bold.

### Cognitive testing

WM was measured using a verbal 2-back task that included familiar lure items (Gray et al. 2003; Marklund and Persson 2012) occurring either at 3, 5, 6, 7, 8, 9, or 10 trial(s) after first item presentation (ie 3- to 10-back lures; Samrani et al. 2017). These are referred to as 3B, 5B etc., up to 10B. The task was divided into two equal blocks of 105 trials each, with a 1-min break between blocks. The task consisted of (i) non-familiar words presented for the first time (new trials), (ii) words presented for the second time at the correct 2B position (target trials), (iii) words presented for the second time at an incorrect position, one word after the target position (3B; proximal lures), and (iv) words presented a third time, three to ten trials from the target position (3B to 10B; distant lures).

Lure trials consisted of stimuli already presented 3 to 10 trials earlier and required a “No” response, and new trials were non-familiar trials that had never been presented previously, which also required a “No” response. Target trials were 2B trials and required a “Yes” response. For each presented word, participants were instructed to press with the right index finger on an MRI-compatible response box, which corresponds to “Yes” (“Yes, the word I now see has been shown two words ago”) and the button on the middle finger for “No” (“No, the word I now see has not been shown two words ago”). Distant lures were all recycled from either previous target trials or proximal lures with the aim to lower the total amount of new trials, consequently the proportion of “No” answers. In the current study, WM performance was based on accuracy and reaction times (RTs) for target trials, and PI in WM was measured using a combined RT and accuracy score for proximal and distant trials.

Stimuli and trial conditions were presented in the same fixed order for all participants and consisted of common Swedish nouns with a maximum of two syllables. Stimuli were presented one at a time for 2.5 s, with a varying inter-trial jittering (2, 2.66, 3.33, or 3.99 s). There was an even distribution of the jitter timings, and the timing from a new trial to the target position always added up to a total inter-trial time of 5.99 s to avoid any differences in encoding time between target items. Participants were instructed to answer as quickly and accurately as possible. Relative difference scores (PI scores) were calculated as the relative proportional difference in RT and accuracy between non-familiar trials and familiar lure trials (Samrani et al. 2017; Samrani et al. 2019). Interference can thus be observed as the difference in % between lure trials (high interference trials) and non-familiar trials (no interference trials). A relative difference score should represent a more salient measure of executive control, as it considers baseline individual differences in the variables in question, such as processing speed. Median RTs were used to reduce the influence of extreme values. PI scores based on RTs and accuracy were positively correlated and therefore combined by calculating the average of the two difference scores.

### Behavioral statistical analyses

Performance on target trials and the measure of PI, representing two different aspects of WM processing, were of particular interest in the current study. RTs were calculated for correct trials only. Median RTs were extracted for target trials, new trials, and lure trials for each condition. The two scores representing PI were normalized with z-score transformation and combined by calculating the average of the two difference scores. Separate scores for proximal lure trials (3B) and distant lure trials (5B to 10B trials) were calculated. PI scores for RT and accuracy were positively correlated (proximal lures: *r*(73) = 0.31, *P* = 0.007; distant lures: *r*(73) = 0.39, *P* = 0.001).

### Magnetic resonance data acquisition

Data were collected on a 3 T GE Signa Premier scanner (General Electric Healthcare, USA) with a 48-channel head coil. Participants were instructed to keep their head and neck stable, stay awake, close their eyes, and relax during the magnetic resonance (MR) scans. The scanning protocol included acquisition of a 3D axial T_1_-weighted BRAin VOlume imaging scan for voxel placement (T1 450 = ms, 1 mm^3^ isotropic voxels, flip angle = 24°). Two volumes of interest (VOIs) were placed in the right inferior frontal gyrus (25 × 35 × 25 mm; 21.875 ml) and right HC (30 × 40 × 20 mm; 24 ml; Fig. 2A). To achieve higher consistency, the voxel was placed for all participants on their T1-weighted image by the same team of operators, avoiding orbital, ventricular, and insular areas. Voxel positioning and volume were chosen as a compromise between the best achievable spectral linewidth and the lowest level of spectral artifacts. The ^1^H spectrum optimized for detecting GABA was acquired individually for this voxel using the MEGA-PRESS sequence with the following parameters: echo time/repetition time = 68/2000 ms, number of points = 2048, spectral width = 2000 Hz, and number of raw averages = 256 (scan time, 9 min 20 s). Since the chemical structure of glutamate results in multiple resonances that overlap with signals from other brain metabolites, we quantified the combined glutamate and glutamine signals (Glx).

### MRS preprocessing and analysis

**Fig. 1:**
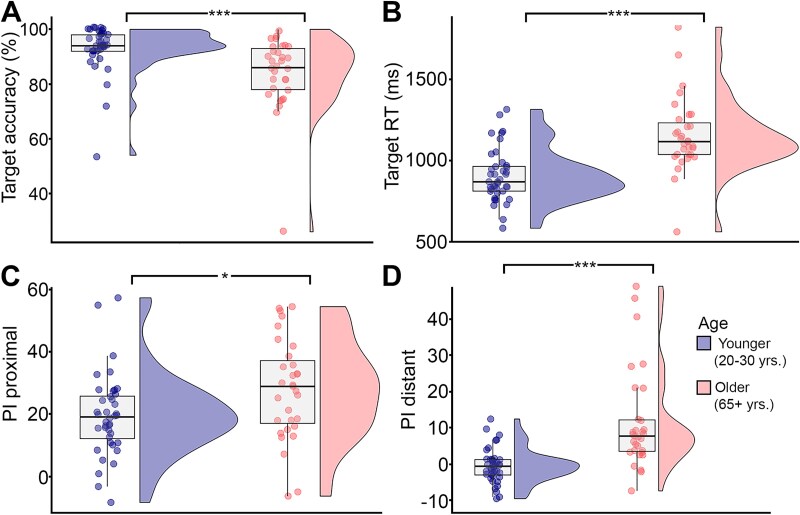
Mean RTs and accuracy for lure trials in the 2-back task for each age group: younger adults and older adults. (A) Target accuracy, (B) target RT, (C) mean interference effect (new negative trial vs. familiar 3-back lure trial) for proximal trials, and ((D) mean interference effect (new negative trial vs. familiar 5- to 10-back lure trials) for distant trials. * < 0.05, *** < 0.005.

MRS data were analyzed using the Osprey software (https://schorschinho.github.io/osprey/) version 2.5.0 (Oeltzschner et al. 2020), an open-source MRS analysis toolbox in MATLAB (R2022a). We used Osprey’s Linear Combination Model to estimate GABA^+^ and Glx. The output GABA^+^ and Glx concentrations were expressed in international units relative to water (GABA^+^ and Glx/water), and the segmented structural (T1) image was used along with a tissue-correction method to account for gray matter, white matter, and cerebrospinal fluid composition of the VOI (Fig. 1B). MRS data were excluded if they demonstrated significant motion artifacts, insufficient water suppression, or major lipid contamination.

### MRS quality indices

MRS quality measures are shown in Table 1. We observed a significant age-related reduction in signal-to-noise (SNR) ratio in the HC (F(1,73) = 6.15, *P* = 0.015, ƞ_p_^2^ = 0.081), but not in the IFG. MRS linewidth (full width at half maximum, FWHM) was comparable between younger and older adults in both HC and IFG (HC: F(1,73) = 0.204, *P* = 0.653, ƞ_p_^2^ = 0.003; IFG: F(1,73) = 0.020, *P* = 0.88, ƞ_p_^2^ < 0.001). Age differences in tissue composition within the HC showed that older adults had less GM ((1,73) = 41.9, *P* < 0.001, ƞ_p_^2^ = 0.378, and WM ((1,73) = 8.24, *P* = 0.005, ƞ_p_^2^ = 0.107) and more CSF ((1,73) = 52.8, *P* < 0.001, ƞ_p_^2^ = 0.433) compared to younger adults. For the IFG, older adults had less GM ((1,73) = 127, *P* < 0.001, ƞ_p_^2^ = 0.65) and more CSF ((1,73) = 51.6, *P* < 0.001, ƞ_p_^2^ = 0.428) compared to younger adults. There was no age difference in IFG WM ((1,73) = 3.65, *P* = 0.06, ƞ_p_^2^ = 0.005).

### Statistical analysis

Shapiro–Wilk testing and subsequent inspection of quantile–quantile (Q-Q) plots indicated that concentration estimates of GABA^+^/Glx in both HC and IFG satisfied the assumption of a normal distribution. For all associations between GABA^+^/Glx and WM, we use residual scores controlling for age, sex, and education. Age differences in behavioral outcomes and GABA^+^/Glx concentration were tested using between-group analysis of variance (ANOVAs). For behavioral analyses, we first analyzed differences between trial types on RT and accuracy and then separate between-group analyses on target RT and accuracy, along with measures of proximal and distant PI (see section Results). All statistical analyses were conducted using SPSS software, and significance was defined at the *P* = 0.05 level. Partial eta squared (ƞ_p_^2^) was used to measure effect size. Moderation (model 1) and mediation (model 4) analyses were performed using the PROCESS macro implemented in SPSS. For moderation, age was used as the moderator in the relationship between GABA^+^/Glx and WM. Moderation analyses were performed only when an association was significant in one of the age groups, but not in the other. For the mediation analyses, age was defined as the independent variable, WM (target accuracy and PI) as the outcome variable, and IFG/HC GABA^+^/Glx as the mediator. Thus, a total of 8 mediator models were tested.

## Results

### Behavioral results

#### Analyses across all participants

*Accuracy:* Across participants, accuracy for target trials (89.4%) was not significantly different from non-familiar trials (91.6%; F(1,73) = 2.69, *P* = 0.105, ƞ_p_^2^ = 0.036) and distant lure trials (89.8%; F(1,73) = 0.437, *P* = 0.511, ƞ_p_^2^ = 0.006), but was higher compared to familiar proximal trials (69%; F(1,73) = 71.3, *P* < 0.001, ƞ_p_^2^ = 0.498). Accuracy for proximal lure trials was lower compared to both non-familiar trials (F(1,73) = 112, *P* < 0.001, ƞ_p_^2^ = 0.61) and distant lure trials (F(1,73) = 137, *P* < 0.001, ƞ_p_^2^ = 0.657). The difference between non-familiar negative trials and distant lure trials was not significant (F(1,73) = 1.36, *P* = 0.247, ƞ_p_^2^ = 0.019).

*Response times:* Across participants, RTs for target trials (1024 ms) were shorter compared to both non-familiar negative trials (1126 ms; F(1,73) = 7.46, *P* = 0.008, ƞ_p_^2^ = 0.094), distant lure trials (1185 ms; F(1,73) = 25.2, *P* < 0.001, ƞ_p_^2^ = 0.259), and proximal lure trials (1377 ms; F(1,73) = 134, *P* < 0.001, ƞ_p_^2^ = 0.651). RTs for proximal lure trials were longer compared to both non-familiar negative trials (F(1,73) = 95.8, *P* < 0.001, ƞ_p_^2^ = 0.571), and distant lure trials (F(1,73) = 79.5, *P* < 0.001, ƞ_p_^2^ = 0.525). In addition, RTs for non-familiar negative trials were shorter compared to distant lure trials (F(1,73) = 10.7, *P* = 0.002, ƞ_p_^2^ = 0.13).

#### Between-group analyses

For target accuracy, older adults had worse performance compared to younger adults (Fig. 1A; F(1,73) = 11.8, *P* = 0.001, ƞ_p_^2^ = 0.144, and older adults also responded more slowly on target trials (Fig. 1B; F(1,73) = 28.8, *P* < 0.001, ƞ_p_^2^ = 0.292). Furthermore, older adults were more affected by the manipulation of PI, as indicated by a statistically higher PI for both proximal (Fig. 1C; F(1,73) = 6.02, *P* = 0.017, ƞ_p_^2^ = 0.079) and distant trials (Fig. 1D; F(1,73) = 14.9, *P* < 0.001, ƞ_p_^2^ = 0.175).

### MRS results

In line with previous studies (Steel et al. 2020), GABA^+^ and Glx levels were positively correlated, both with (HC: r(73) = 0.31, *P* = 0.01; IFG: r(73) = 0.33, *P* = 0.004) and without (HC: r(73) = 0.3, *P* = 0.012; IFG: r(73) = 0.31, *P* = 0.007) controlling for age, sex, and education.

#### Age differences in Glx and GABA between younger and older individuals

**Fig. 2:**
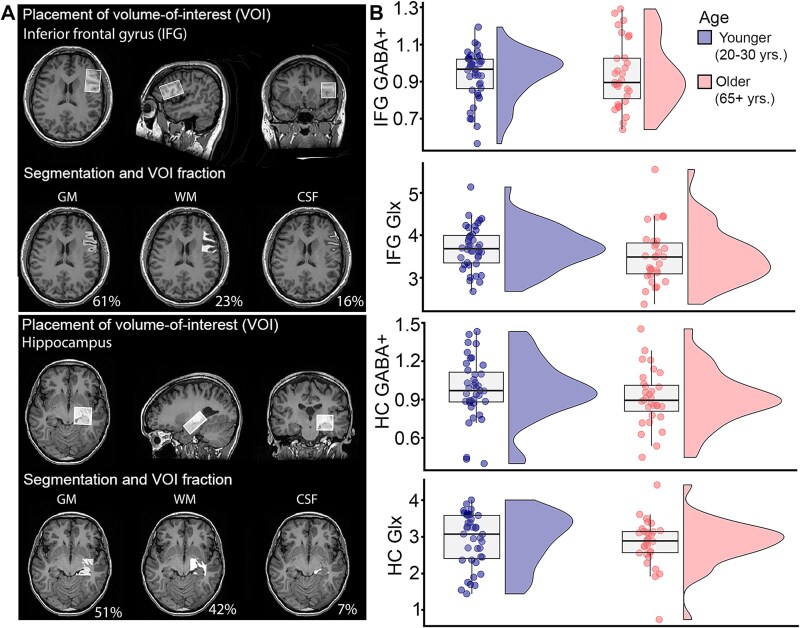
(A) Placement of each VOI in the inferior frontal gyrus (top) and hippocampus (bottom) along with examples of tissue composition within the VOI. (B) Average tissue-corrected signal intensity of metabolites relative to tissue water for younger and older individuals.

A between-group ANOVA showed that there were no significant differences between younger and older adults in tissue-corrected Glx (Fig. 2B; HC: F(1,73) = 0.25, *P* = 0.619, ƞ_p_^2^ = 0.004; IFG:: F(1,73) = 2.35, *P* = 0.13, ƞ_p_^2^ = 0.033) or GABA (HC: F(1,73) = 1.57, *P* = 0.214, ƞ_p_^2^ = 0.022; IFG:: F(1,73) = 0.063, *P* = 0.803, ƞ_p_^2^ = 0.001) measurements. Including individual variability in signal quality (SNR, fit error, and FWHM) did not change these results.

#### Associations between WM measurements and Glx/GABA

Since both distant and proximal lure trials had a similar age effect, and in order to reduce the number of comparisons, we collapsed between these two trial types in the analyses of associations between WM and GABA/Glx. Bivariate correlations for all variables are shown in Supplementary Tables S1-S3.

#### Analyses across all participants

**Fig. 3:**
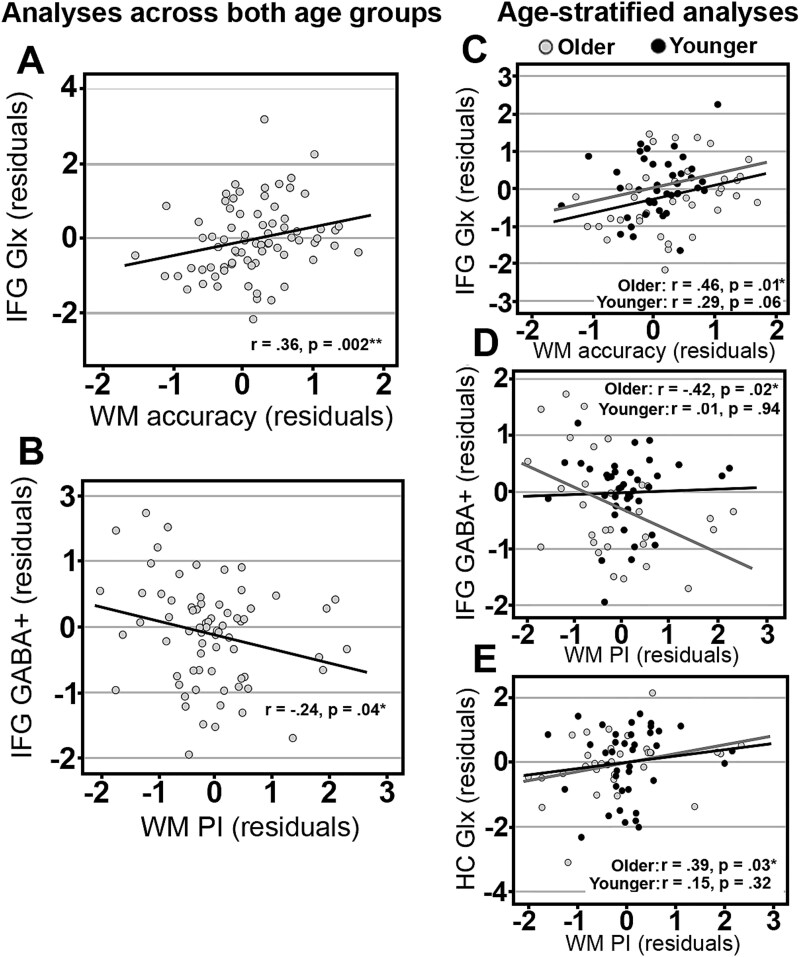
Scatter plots of simple regressions between GABA+/Glx and WM component processes (residuals adjusted for age and sex). IFG = inferior frontal gyrus, HC = hippocampus, PI = proactive interference.

Across all participants, we found a positive correlation between WM accuracy for target trials and IFG Glx (Fig. 3A; r(73) = 0.355, *P* = 0.002). There was also a significant negative correlation between PI and IFG GABA^+^ (Fig. 3B; r(73) = −.24, *P* = 0.043). Bivariate correlations for all associations are shown in Supplementary Table S1.

#### Age-stratified analyses

Bivariate correlations for all associations are shown in Supplementary Tables S2 and S3. In older adults, there was a significant positive correlation between WM accuracy for target trials and IFG Glx (Fig. 3C; *r*(31) = 0.455, *P* = 0.01), while this association was not significant for younger adults (*r*(42) = 0.285, *P* = 0.061). A moderation analysis showed that age was not a significant moderator in the relationship between WM accuracy for target trials and IFG Glx (F = 0.543, *P* = 0.464, lower-level confidence interval (LLCI) = −.073, upper-level confidence interval (ULCI) = 0.159). In older adults, there was a significant negative association between PI and IFG GABA^+^ (Fig. 3D; *r*(31) = −.419, *P* = 0.019), and a significant positive correlation between PI and HC Glx (Fig. 3E; *r*(31) = 0.39, *P* = 0.03). These correlations were not significant in younger adults (IFG GABA^+^: *r*(42) = 0.012, *P* = 0.94; HC Glx: *r*(41) = 0.154, *P* = 0.324). Moderation analyses showed that age was not a significant moderator in these relationships (PI—IFG GABA^+^: F = 2.61, *P* = 0.111, LLCI = −.018, ULCI = 0.002; PI—HC Glx: F = 2.11, *P* = 0.151, LLCI = −.002, ULCI = 0.014).

### Mediation results

Mediation analyses were conducted to examine whether GABA^+^/Glx concentrations were significant mediators of the association between age and WM. However, these mediation analyses were all no-significant, indicating that GABA^+^/Glx concentrations did not mediate the relationship between age and WM. The results from the mediation analyses are found in Supplementary Table S4.

## Discussion

In the current study, we aimed to assess the relationship between component processes of WM and GABA^+^ and Glx in younger and older adults. While older adults performed worse on all measures of WM, no age differences in tissue-corrected estimates of GABA^+^ and Glx were found. Across all participants, we found a significant positive association between WM accuracy and Glx in the IFG, as well as a significant negative association between IFG GABA+ and PI. No other relationships between GABA^+^/Glx and WM were found across all participants. Age-stratified analyses demonstrated a significant positive association between PI and HC Glx, as well as a significant negative association between IFG GABA^+^ and PI in older adults only. These results provide novel evidence for the specific involvement of excitatory Glx in WM accuracy and inhibitory GABA^+^ in the control of PI in WM, and how these effects are differentially affected by age.

Contrary to many previous studies (Marenco et al. 2018; Roalf et al. 2020; Porges et al. 2021), we did not find a significant age difference in IFG or HC GABA^+^/Glx. This may not be too surprising, however, given that many recent studies have demonstrated that age differences in neurochemical concentration are largely driven by tissue composition within the VOI (Porges et al. 2017b; Maes et al. 2018). It has been repeatedly demonstrated that measures of metabolites using MRS are heavily influenced by the composition of white and gray matter as well as CSF within the VOI (Harris et al. 2015; Porges et al. 2017b), with a higher distribution of GABA in gray matter compared to white matter. Since tissue composition differs between younger and older adults, age differences can largely be accounted for by differences in tissue composition. Therefore, reduced GABA^+^/Glx concentrations during both pathological and normal aging are most likely linked to gray matter atrophy and demyelination, and correcting for tissue composition would effectively remove the main source of this age difference.

Consistent with much previous evidence (Salthouse and Babcock 1991; Braver and West 2008), older adults both responded more slowly and performed at a lower level compared to younger adults on WM target trials. Across both age groups, target accuracy was positively correlated with IFG Glx, which corroborates previous findings of glutamate concentration in the parietal cortex (Zacharopoulos and Kadosh Cohen 2021) and the dorsolateral prefrontal cortex (Oh et al. 2024) in younger adults. Additionally, it was recently found that glutamate in the medial frontal gyrus was negatively associated with WM (ie more glutamate was related to less WM decay) across both younger and older adults. Taken together, these results are in line with the view of WM as dependent on persistent activation of neural firing in collectively tuned neurons within recurrent excitatory networks in the prefrontal (and parietal) cortices (Goldman-Rakic 1995; Andersen and Buneo 2002). Within these networks, glutamatergic signaling more generally, and N-methyl-D-aspartate (NMDA) receptor activation more specifically, is suggested to underlie successful maintenance of activity over the WM delay period (Durstewitz et al. 2000; Van Vugt et al. 2020).

A relationship between GABA and higher-level cognition—including WM and executive control—has been demonstrated previously (Li et al. 2022). While no study to date has examined proactive control directly, our results are well in line with previous observations of inhibitory GABA as critical for controlling access to task-relevant information while simultaneously avoiding interference from nonrelevant information. For example, using the think/no-think paradigm, it has been reported that HC, but not frontal, GABA concentration predicts inhibitory control over unwanted thoughts (Schmitz et al. 2017). In addition, in the dorsolateral prefrontal cortex, GABA concentration has been linked to WM load capacity (Yoon et al. 2016). This study extends previous findings by showing that the ability to control interference in WM is associated with inhibitory neurochemical properties within the IFG, a region known to be involved in interference control. Moreover, this association was found only in older adults. The finding of stronger brain-WM associations in older adults is consistent with many previous studies across multiple brain structural and functional brain modalities (Pläschke et al. 2020; Andersson et al. 2022; Andersson et al. 2023). While no age differences were found in GABA in this region, these findings suggest that older adults are more reliant on excitatory and inhibitory neurotransmission for optimal WM performance, and that these associations only emerge in older age when these neural resources begin to become limited.

There are multiple ways in which GABA^+^ can influence WM functions. It has been shown that GABAergic neurotransmission can modulate pyramidal neuron activity into synchronized gamma oscillations (Whittington and Traub 2003; Cardin et al. 2009). Gamma oscillations are fundamental to higher-order cognition, including WM (Meltzer et al. 2008), and there is a link between cortical GABA concentration and gamma oscillations in humans (Chen et al. 2014). Moreover, findings from a recent multimodal imaging study (Kujala et al. 2024) suggest that GABAergic inhibition is critical for shaping both gamma oscillations and brain activation in cortical regions and its relationship to individual differences in WM performance. Another way in which GABA might influence—which also is relevant for our differential finding in younger and older adults—is through the well-established link between GABAergic and cholinergic systems. Since the cholinergic system is altered in normal and pathological aging (Schliebs and Arendt 2011) and is central for learning and memory (Deutsch 1971), both systems might—independently, or through their interaction—contribute to WM performance.

While associations between GABA^+^/Glx differed between younger and older adults, formal moderation models did not support that these relationships were statistically different between the groups. Therefore, the interpretation of group differences in these associations should be interpreted cautiously. Moreover, mediation analyses were unable to confirm that GABA^+^ or Glx mediated the observed relationships between age and WM component processes. While our sample size is on par with many other MRS studies, mediation analysis typically requires very large samples to ensure reasonable power levels (Fritz and Mackinnon 2007). Relying on a noisy proxy measurement such as GABA^+^ or Glx is also potentially affecting the interpretation of mediation analyses (Westfall and Yarkoni 2016) and has an additional limitation that it cannot capture the hypothesized mediating factor directly. An alternative explanation, therefore, could be that age really is a causal factor of both metabolic changes in glutamate and, separately, cognitive decline in WM component processes. The current study cannot conclusively separate between these possibilities.

One limitation of the current study is the potential contribution of macromolecules to the MRS signal. Given that macromolecules have been shown to increase with aging (Hofmann et al. 2001; Marjańska et al. 2018), the effect of age on GABA^+^ and Glx might be underestimated in the present study. Another limitation is that while MRS allows for quantification of brain metabolism noninvasively, it remains unclear how to interpret the resulting signals regarding its underlying cellular mechanisms. For example, it is not known if it reflects the entire pool of GABA/Glx available for measurement (ie intracellular, extracellular, and metabolic) or if it reflects mostly extracellular extrasynaptic levels not directly related to synaptic transmission (Stokes et al. 2014; Lea-Carnall et al. 2023). A final limitation is that we did not obtain GABA/Glx measurements from a control region without a proposed involvement in WM. While we hypothesize somewhat differential involvement, both in terms of region specificity (IFG vs. HC) and neurotransmitter specificity (GABA vs. Glx) in WM performance and WM interference control, the lack of control regions makes interpretation of these regions as critical for WM less specific.

Here, we demonstrate that inferior frontal Glx is positively related to WM target accuracy across both younger and older individuals. This suggests that maintaining representations in WM critically depends on excitatory signaling sustained by the glutamatergic system. Furthermore, frontal GABA^+^ was related to less PI in WM across both age groups, and this association was more pronounced in older adults. This provides evidence for the inhibitory role of GABA in the control of WM. In addition, in older adults only, HC Glx was associated with more PI, possibly indicating that excitatory Glx signaling strengthens WM representation, making them more resilient to interference control. Taken together, these findings suggest a potential age-modulatory role of Glx and GABA^+^ in HC and frontal regulation of WM.

## Supplementary Material

AnderssonP_Supplementary_Tables_bhaf105
