# Supplementary material for: Hippocampal and prefrontal GABA and glutamate concentration contribute to component processes of working memory in aging
Source: Cereb Cortex. 2025 May 12;35(5):bhaf105. doi: 10.1093/cercor/bhaf105 (PMC12066406; doi:10.1093/cercor/bhaf105)
Supplement: AnderssonP_Supplementary_Tables_bhaf105 [file anderssonp_supplementary_tables_bhaf105.docx]

Supplementary materials

Hippocampal and prefrontal GABA and Glx concentration contribute to component processes of working memory in aging.

Pernilla Andersson^1^, Xin Li^2,3^, and Jonas Persson^1, 2^

^1^Center for Life-span Developmental Research (LEADER), School of Behavioral, Social and Legal sciences, Örebro University, Sweden ^2^Aging Research Center (ARC), Karolinska Institute and Stockholm University, Sweden ^3^Computational Brain Imaging Group, National University of Singapore, Singapore

Running title: GABA and Glx concentration, working memory and aging.

*Corresponding author: Jonas Persson, Center for Life-span Developmental Research (LEADER), School of Behavioral, Social and Legal sciences, Örebro University Fakultetsgatan 1, 702 81, Örebro, Sweden E-mail: jonas.persson.1@ki.se

Supplementary Table 1. Bivariate correlations among the study variables across all participants.

|  | 1 | 2 | 3 | 4 | 5 | 6 | 7 |
| --- | --- | --- | --- | --- | --- | --- | --- |
| 1. HC Glx | 1 |  |  |  |  |  |  |
| 2. HC GABA+ | .310** | 1 |  |  |  |  |  |
| 3. IFG Glx | .165 | .038 | 1 |  |  |  |  |
| 4. IFG GABA+ | .053 | .242* | .330** | 1 |  |  |  |
| 5. WM Target accuracy | .003 | -.139 | .355** | -.048 | 1 |  |  |
| 6. WM Target RT | .005 | -.033 | -.215 | -.026 | -.408*** | 1 |  |
| 7. WM Proactive interference | .215 | .111 | -096 | -.24* | -.149 | .033 | 1 |
| **p*<.05, ***p*<.01, ****p*<.005 | | | | | | | |

Supplementary Table 2. Bivariate correlations among the study variables in the group of younger adults.

|  | 1 | 2 | 3 | 4 | 5 | 6 | 7 |
| --- | --- | --- | --- | --- | --- | --- | --- |
| 1. HC Glx | 1 |  |  |  |  |  |  |
| 2. HC GABA+ | .260 | 1 |  |  |  |  |  |
| 3. IFG Glx | .264 | .007 | 1 |  |  |  |  |
| 4. IFG GABA+ | .133 | .362* | .291 | 1 |  |  |  |
| 5. WM Target accuracy | -.005 | -.090 | .285 | .236 | 1 |  |  |
| 6. WM Target RT | -.046 | .001 | -.235 | .040 | -.385* | 1 |  |
| 7. WM Proactive interference | .154 | .155 | -.212 | .012 | -.495** | .124 | 1 |
| **p*<.05, ***p*<.01, ****p*<.005 |  |  |  |  |  |  |  |

Supplementary Table 3. Bivariate correlations among the study variables in the group of older adults.

|  | 1 | 2 | 3 | 4 | 5 | 6 | 7 |
| --- | --- | --- | --- | --- | --- | --- | --- |
| 1. HC Glx | 1 |  |  |  |  |  |  |
| 2. HC GABA+ | .374* | 1 |  |  |  |  |  |
| 3. IFG Glx | .165 | .147 | 1 |  |  |  |  |
| 4. IFG GABA+ | .060 | .265 | .428** | 1 |  |  |  |
| 5. WM Target accuracy | .038 | .037 | .455** | .100 | 1 |  |  |
| 6. WM Target RT | .007 | -.079 | -.130 | -,007 | -.441** | 1 |  |
| 7. WM Proactive interference | .390* | .230 | .004 | -.419* | -.210 | .008 | 1 |

**p*<.05, ***p*<.01, ****p*<.005

Supplementary Table 4. Results from mediation analyses.

| Independent variable (X) | Dependent variable (Y) | Mediator (M) | Effect (B) | SE | 95% CI (LL) | 95% CI (UL) |
| --- | --- | --- | --- | --- | --- | --- |
| Age | WM target accuracy | HC GABA^+^ | -.001 | .006 | -,011 | .015 |
| Age | WM target accuracy | HC Glx | ,000 | ,004 | -,009 | ,009 |
| Age | WM target accuracy | IFG GABA^+^ | ,001 | ,005 | -,011 | ,012 |
| Age | WM target accuracy | IFG Glx | -,005 | ,010 | -,026 | ,016 |
| Age | WM Proactive interference | HC GABA^+^ | -,011 | ,017 | -,057 | ,006 |
| Age | WM Proactive interference | HC Glx | -,005 | ,020 | -,049 | ,034 |
| Age | WM Proactive interference | IFG GABA^+^ | ,001 | ,011 | -,020 | ,027 |
| Age | WM Proactive interference | IFG Glx | -,002 | ,012 | -,024 | ,029 |

SE = standard deviation, CI = bootstrapped confidence intervals, LL = lower limit of CI, UL = upper limit of CI.
